# Supplementary material for: Modeling glioblastoma heterogeneity as a dynamic network of cell states
Source: Mol Syst Biol. 2021 Sep 16;17(9):e10105. doi: 10.15252/msb.202010105 (PMC8444284; doi:10.15252/msb.202010105)
Supplement: Supplementary file 6 — Source Data for Figure 5 [file MSB-17-e10105-s004.zip › Figure5A_sourcedata/GSEA_3071/hallmarks_stateII.GseaPreranked.1623742536692/HALLMARK_KRAS_SIGNALING_DN.html]

Details for gene set HALLMARK\_KRAS\_SIGNALING\_DN[GSEA]

|  || Dataset | state23071 |
| Phenotype | NoPhenotypeAvailable |
| Upregulated in class | na\_neg |
| GeneSet | HALLMARK\_KRAS\_SIGNALING\_DN |
| Enrichment Score (ES) | -0.36855528 |
| Normalized Enrichment Score (NES) | -1.50877 |
| Nominal p-value | 0.05586592 |
| FDR q-value | 0.16946419 |
| FWER p-Value | 0.74 |
Table: GSEA Results Summary

  

Fig 1: Enrichment plot: HALLMARK\_KRAS\_SIGNALING\_DN      
 Profile of the Running ES Score & Positions of GeneSet Members on the Rank Ordered List

  

| PROBE | GENE SYMBOL | GENE\_TITLE | RANK IN GENE LIST | RANK METRIC SCORE | RUNNING ES | CORE ENRICHMENT || 1 | EDN1 |  |  | 6 | 1.670 | 0.0832 | No |
| 2 | BARD1 |  |  | 295 | 0.760 | 0.0510 | No |
| 3 | SLC16A7 |  |  | 396 | 0.681 | 0.0610 | No |
| 4 | RIBC2 |  |  | 460 | 0.646 | 0.0783 | No |
| 5 | TENM2 |  |  | 555 | 0.599 | 0.0856 | No |
| 6 | COPZ2 |  |  | 793 | 0.501 | 0.0528 | No |
| 7 | GPR19 |  |  | 1261 | 0.385 | -0.0424 | No |
| 8 | GTF3C5 |  |  | 1469 | 0.344 | -0.0758 | No |
| 9 | EDN2 |  |  | 1588 | 0.326 | -0.0883 | No |
| 10 | SGK1 |  |  | 1806 | 0.298 | -0.1264 | No |
| 11 | MSH5 |  |  | 1944 | 0.280 | -0.1459 | No |
| 12 | EFHD1 |  |  | 2224 | -0.250 | -0.2017 | No |
| 13 | YPEL1 |  |  | 2589 | -0.299 | -0.2760 | No |
| 14 | CLSTN3 |  |  | 2604 | -0.301 | -0.2641 | No |
| 15 | SNN |  |  | 2873 | -0.349 | -0.3123 | No |
| 16 | CCDC106 |  |  | 3103 | -0.400 | -0.3483 | Yes |
| 17 | GAMT |  |  | 3145 | -0.413 | -0.3374 | Yes |
| 18 | DCC |  |  | 3155 | -0.415 | -0.3186 | Yes |
| 19 | CNTFR |  |  | 3176 | -0.422 | -0.3021 | Yes |
| 20 | SLC25A23 |  |  | 3348 | -0.475 | -0.3200 | Yes |
| 21 | PTPRJ |  |  | 3519 | -0.553 | -0.3337 | Yes |
| 22 | SKIL |  |  | 3531 | -0.560 | -0.3080 | Yes |
| 23 | CELSR2 |  |  | 3550 | -0.570 | -0.2835 | Yes |
| 24 | TGFB2 |  |  | 3774 | -0.766 | -0.2995 | Yes |
| 25 | TFAP2B |  |  | 3800 | -0.789 | -0.2656 | Yes |
| 26 | BTG2 |  |  | 3917 | -0.999 | -0.2435 | Yes |
| 27 | KCNQ2 |  |  | 3927 | -1.014 | -0.1942 | Yes |
| 28 | KLHDC8A |  |  | 4005 | -1.301 | -0.1471 | Yes |
| 29 | IGFBP2 |  |  | 4094 | -3.345 | 0.0010 | Yes |
Table: GSEA details [plain text format]

  

Fig 2: HALLMARK\_KRAS\_SIGNALING\_DN: Random ES distribution      
 Gene set null distribution of ES for **HALLMARK\_KRAS\_SIGNALING\_DN**
